# Self-Patterned Stretchable Electrode Based on Silver Nanowire Bundle Mesh Developed by Liquid Bridge Evaporation

**DOI:** 10.3390/nano11112865

**Published:** 2021-10-27

**Authors:** Eun Young An, Siyoung Lee, Seung Goo Lee, Eunho Lee, Jeong Ju Baek, Gyojic Shin, Kyung Ho Choi, Jeong Ho Cho, Geun Yeol Bae

**Affiliations:** 1 Green and Sustainable Materials R&D Department, Korea Institute of Industrial Technology, Cheonan 31056, Korea; dksdmsdud97@kitech.re.kr (E.Y.A.); baekjj@kitech.re.kr (J.J.B.); gyshin@kitech.re.kr (G.S.); khchoi@kitech.re.kr (K.H.C.); 2 Department of Chemical and Biomolecular Engineering, Yonsei University, Seoul 03722, Korea; 3 Department of Chemical Engineering, Pohang University of Science and Technology, Pohang 37673, Korea; challenge@postech.ac.kr; 4 Department of Chemistry, University of Ulsan, Ulsan 44610, Korea; lees9@ulsan.ac.kr; 5 Department of Chemical Engineering, Kumoh National Institute of Technology, Gumi 39177, Korea; leeeh@kumoh.ac.kr

**Keywords:** stretchable electrode, self-patterning, liquid bridge, silver nanowire, spray coating

## Abstract

A new strategy is required to realize a low-cost stretchable electrode while realizing high stretchability, conductivity, and manufacturability. In this study, we fabricated a self-patterned stretchable electrode using a simple and scalable process. The stretchable electrode is composed of a bridged square-shaped (BSS) AgNW bundle mesh developed by liquid bridge evaporation and a stretchable polymer matrix patterned with a microcavity array. Owing to the BSS structure and microcavity array, which effectively concentrate the applied strain on the deformable square region of the BSS structure under tensile stretching, the stretchable electrode exhibits high stretchability with a low ΔR/R_0_ of 10.3 at a strain of 40%. Furthermore, by exploiting the self-patterning ability—attributable to the difference in the ability to form liquid bridges according to the distance between microstructures—we successfully demonstrated a stretchable AgNW bundle mesh with complex patterns without using additional patterning processes. In particular, stretchable electrodes were fabricated by spray coating and bar coating, which are widely used in industry for low-cost mass production. We believe that this study significantly contributes to the commercialization of stretchable electronics while achieving high performance and complex patterns, such as stretchable displays and electronic skin.

## 1. Introduction

Over the past decade, stretchable electronics have received significant attention because of their high potential for a wide range of human-friendly electronics applications, such as skin-attachable healthcare systems [1,2], wearable displays [3,4,5], and implantable devices [6,7,8]. Among the fundamental components of stretchable electronics, the fabrication of stretchable electrodes capable of maintaining their initial electrical conductivity under various modes of mechanical deformation remains a crucial research area. High stretchability, conductivity, mechanical durability, and low processing cost are essential to realizing stretchable electrodes [9]. Accordingly, many studies based on structural approaches [10] and material approaches have been reported [11,12,13]. To improve stretchability, novel structures suitable for mechanical deformation, such as serpentine structures [6,14], mesh-shaped structures [15,16], out-of-plane buckled structures [17,18], and kirigami structures [19,20] have been proposed. Although stretchable electrodes with deformable structures have exhibited high stretchability with minor resistance changes under large deformation, metal deposition under high vacuum and prestraining of the polymer substrate in their fabrication processes make mass production difficult, which is a critical requirement for commercialization and practical applications.

Low-dimensional carbon materials and metal nanomaterials [12,21,22] have been proposed to overcome these challenges. Among them, silver nanowires (AgNWs) have been widely used because of their high conductivity and flexibility induced by thinness [23,24]. In particular, AgNWs wrapped by polyvinylpyrrolidone are well dispersed in polar solvents such as water and alcohols [25]; thus, they have been readily applied to stretchable electrodes through solution processes that allow for straightforward mass production at low cost [26,27]. However, most AgNW-based stretchable electrodes fabricated by simple and scalable solution processes, such as spray and bar coating, exhibit limited stretchability because of the absence of an efficient deformable structure for delocalization and relaxation of localized stress for AgNWs under large deformations. Recently, a stretchable electrode based on a AgNW bundle with a microscaled random mesh form was reported [28,29]. The random mesh was formed by the coffee-ring effect-driven assembly of nanowires under spray coating. Owing to the deformability of the random mesh structure, the electrode exhibited a negligible increase in resistance against a considerable strain. Nevertheless, considering that a sophisticated pattern is essential for the application to stretchable displays, e-skin, and health-monitoring applications that are composed of multiple cells with complex device structures, there is a significant disadvantage in that additional photolithography or laser ablation for electrode patterning must be performed [30].

In this paper, we present a self-patterned stretchable electrode composed of a bridged square-shaped (BSS) AgNW bundle mesh embedded in a stretchable polymer substrate patterned with a microcavity array. By inducing a concave liquid bridge of AgNW dispersion between microstructures upon spray coating, which is widely used in industry for low-cost mass production, we obtained a regular and uniform BSS AgNW bundle mesh favorable to mechanical deformation. In particular, by inducing a microcavity array that was patterned inside the square region of the BSS bundle mesh, we achieved a stretchable electrode that exhibited high stretchability with a low ∆R/R0 of 10.3 at 40% strain. Furthermore, by exploiting the self-patterning ability based on the difference in the ability to form liquid bridges according to the distance between microstructures, we successfully demonstrated a stretchable AgNW bundle mesh with complex patterns without using additional patterning processes. We believe that this study significantly contributes to the commercialization of stretchable electronics with high performance and complex patterns, such as a stretchable antenna for skin attachable RFID patch [31] and battery-free electronic skin powered by Wi-Fi signal [32], and interconnectors of stretchable display with micro LEDs [10].

## 2. Materials and Methods

The microstructured substrates with the dimension of 30 mm × 30 mm were prepared by demolding a cured PDMS (Sylgard 184, Dow Corning, Midland, MI, USA) sheet from a prepatterned Si mold with an inversed micropyramidal cavity array [33]. On the substrate, pyramidal microstructures with the individual dimension of 10 μm × 10 μm (height ~7 μm) were patterned. To investigate the effect of the surface energy of the substrate on the formation of the mesh, fluorinated SAM-treated PDMS by chemical vapor deposition with trichloro(1H,1H,2H,2H-perfluorooctyl)silane (FOTS, Sigma Aldrich, St. Louis, MO, USA) [34] and UVO-treated PDMS by UV exposure in the air using an ozone cleaner (UVC-20, Japan Engineering Co., Hyogo, Japan) were also prepared. On the microstructured substrates, AgNW dispersion (Flexiowire2020c, D 25 nm L 25 μm, 0.5 wt%, SG Flexio Co., Ltd., Daejeon, Korea) diluted to 0.01 wt% in isopropyl alcohol (IPA, Sigma Aldrich) was sprayed using an automatic spray coater (ReVo-S) equipped with a nozzle (AM45, ATOMAX) with a diameter of 1 mm. The spray rate (i.e., the volume of sprayed dispersion per second), pressure, and distance from the nozzle tip to target substrates were 60 μL/s, 0.1 MPa, and 10 cm, respectively. After bar-coating with thermoplastic polyurethane (TPU, MIRATHANE E190, Miracll Chemical Co., Ltd., Yantai, China) solution dissolved in dimethylformamide (Sigma Aldrich) to 10 wt% and dried at 60 °C for 2 h in a vacuum, the stretchable electrode was gently peeled off using an adhesive PET frame. The deposition behavior of AgNWs was observed in situ using an inverted optical microscope (XDS 3FL4, Optika, Ponteranica, Italy) during spray coating. The morphologies after AgNW deposition or subsequently applied tensile strain were observed using an optical microscope (VK-X1000, KEYENCE) and FE-SEM (JSM-6701F, JEOL). The surface-modified substrates were characterized by X-ray photoelectron spectroscopy (XPS, K-Alpha, Thermo Fisher, Waltham, MA, USA) and a contact angle analyzer (SmartDrop Plus, Femtobiomed, Seongnam, Korea). Relative resistance changes (ΔR/R_0_) were analyzed using an I-V meter (4200A-SCS, Keithley, Tacoma, WA, USA), and the sheet resistances were measured using a four-point probe module (PE100, MSTECH, Bath, PA, USA) connected to the I-V meter. The stress distribution on the BSS bundle mesh under 30% tensile strain was analyzed using finite element method-based simulation (COMSOL Multiphysics^®^ 5.2a). A solid mechanics module and nonlinear structural material module were utilized to stretch the AgNW bundle mesh and the TPU substrate. The result of the stress distribution was expressed as the von Mises stress (N/m) applied to the AgNW bundle mesh.

## 3. Results and Discussion

**Figure 1:**
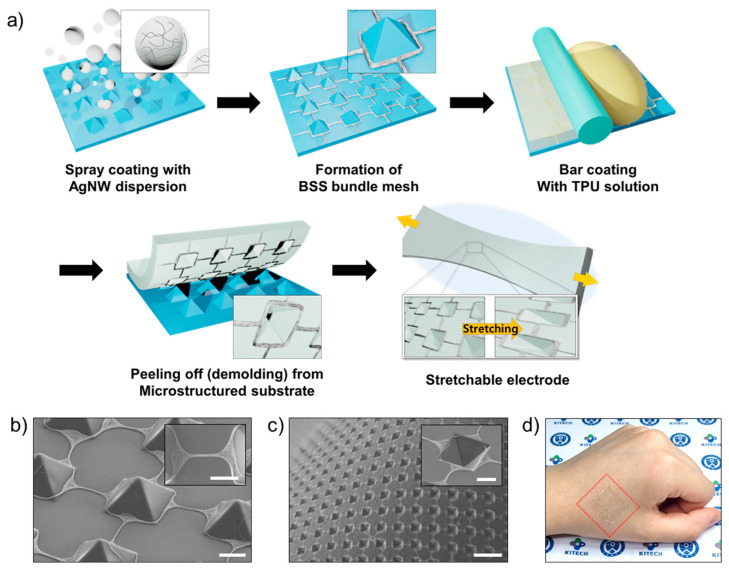
(**a**) Schematic of the fabrication process of the stretchable electrode. (**b**) FE-SEM images of the BSS bundle mesh after spray coating on microstructured PDMS substrate. The inset is a high-magnification image of the bridge region in the BSS mesh, where AgNW is well-aligned and packed. Scale bar: 5 μm. (**c**) FE-SEM image of the stretchable electrode after peeling off from the microstructured substrate of Figure 1b. Scale bar: 30 μm. The inset is a high-magnification image of a microcavity located inside a square region of the BSS mesh. Scale bar: 5 μm. (**d**) Image of the stretchable electrode conformally attached on the back of a human hand.

The self-patterned stretchable electrode was fabricated simply, as shown in Figure 1a. Detailed information on the fabrication of the stretchable electrode is described in Section 2. As it is fabricated by fabrication processes common to industry, that is, spray and bar coating, scalable and continuous production is feasible. Even if a PDMS substrate with a micropyramidal structure array is fabricated by a batch-type process such as lithography and etching, it can be semipermanently reusable. By spray coating the microstructured substrate with AgNW dispersion, a BSS bundle mesh was developed, as shown in Figure 1b. Interestingly, very few AgNWs were deposited on the surface of the micropyramidal structure. Most of the AgNWs were placed at the contact line between the microstructure and the basal plane (square region) and between the microstructures with a well-ordered form (bridge region). After bar coating the TPU solution and drying, the stretchable electrode was obtained by being peeled off from the microstructured PDMS substrate. The AgNW mesh was successfully transferred from the top surface of the microstructured PDMS substrate to the TPU matrix in an embedded form. In addition, an inversed pyramidal microcavity array was naturally patterned inside the square region of the AgNW mesh by demolding (Figure 1c). Owing to its thinness and stretchability, the stretchable electrode can be conformally attached to curved surfaces such as human skin. In addition, it was translucent (Figure 1d), allowing the stretchable electrode to be unnoticeable when attached to human skin as an electronic skin device.

**Figure 2:**
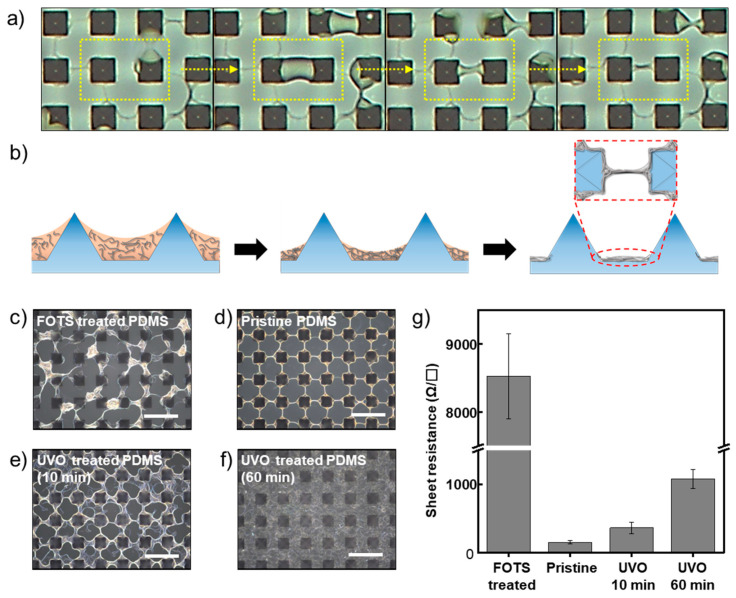
(**a**) In situ captured images during AgNW deposition by spray coating. (**b**) Schematic of the potential formation mechanism of the BSS bundle mesh. (**c**–**f**) Images of deposited AgNW on microstructured substrates with various surface energy. Scale bar: 30 μm. (**c**) FOTS-treated PDMS; (**d**) pristine PDMS; (**e**) UVO (10 min) treated PDMS; (**f**) UVO (60 min) treated PDMS. (**g**) Sheet resistances of the AgNW-deposited substrates are shown in Figure 2c–f. The sheet resistance is the mean value of 5 samples, and the error bar indicates standard deviation.

To study the formation mechanism of the BSS bundle mesh, an in-situ observation of the deposition behavior was conducted using an inverted optical microscope during spray coating. As shown in Figure 2a, the microdroplet was first deposited on the microstructured substrate by spray coating. Then, the deposited droplets moved to and coalesced at the contact line between the microstructure and base surface by the air blown from the nozzle. The coalesced liquids placed outside the individual microstructure were interconnected in a bridge form by successive spray coating. Meanwhile, the interconnecting bridges composed of AgNW dispersion, called a liquid bridge, were developed according to the nearest-bridging rule [35]. After evaporation of the liquid, a BSS bundle mesh was finally formed. From the results of the in situ observation, we conclude that the formation of the BSS bundle mesh results from the evaporation of the liquid bridge and is based on the mechanism of evaporative lithography. Vakarelski et al. reported using evaporative lithography, which exploits various effects generated by solvent evaporation, to fabricate microwire networks and patterns [36]. After dropping the gold nanoparticle dispersion on the lyophilic particulate templates, the liquid bridge started to develop during evaporation. As evaporation proceeded, the shrinking pendular rings formed nodes at the base of the particles joined by thin liquid bridges that were in contact with the substrate [37]. With further evaporation and narrowing of the liquid bridges, the nanoparticles in the liquid bridge were forced to pack in one direction. In our study, a concave liquid bridge was formed between neighboring microstructures made of PDMS with moderately lyophilic properties to the IPA solvent of the dispersion. With the evaporation of the dispersion, narrowing of the liquid bridges occurred, and the AgNWs in the dispersion were aligned and packed. Consequently, the BSS bundle mesh was formed uniformly, as shown in Figure 2b.

To study the effect of the surface energy of the microstructured substrate on the formation of the BSS bundle mesh, microstructured substrates with various surface energies were spray coated. As shown in Figure 2c–f, in the case of FOTS-treated PDMS with lower surface energy compared to pristine PDMS [34], the bundle mesh structure was not formed, and the coating was nonuniform; thus, macroscaled aggregations were observed (Figure 2c). We attribute this result to the microdroplets, deposited on the substrate surface, moving too readily under airflow from the spray nozzle, thus coalescing with each other due to the low interaction with the FOTS-treated PDMS substrate. For the PDMS treated by UVO for 10 min (Figure 2e), the AgNW mesh structure appeared, but the AgNWs were not well aligned and packed. For the PDMS treated by UVO for 60 min (Figure 2f), AgNWs were deposited over the entire surface of the substrate, including the surface of the microstructure (Figure S1). This result is due to the full wetting of microdroplets under spraying, and pinning during evaporation [38]. All the surface-modified substrates without microstructures were characterized by XPS and contact angle analysis, as shown in Figures S2 and S3.

In contrast to substrates treated by FOTS or UVO, the BSS bundle mesh structure was clearly formed for the microstructured PDMS substrate without any surface treatment (Figure 2d). In addition, by virtue of a well-formed mesh structure with a packed and aligned AgNW bundle, it exhibited an even lower sheet resistance of 151 Ω/□ compared to other substrates, as shown in Figure 2g. Although there were some differences according to the spray conditions, such as temperature, pressure, and spraying rate, the trends were similar.

**Figure 3:**
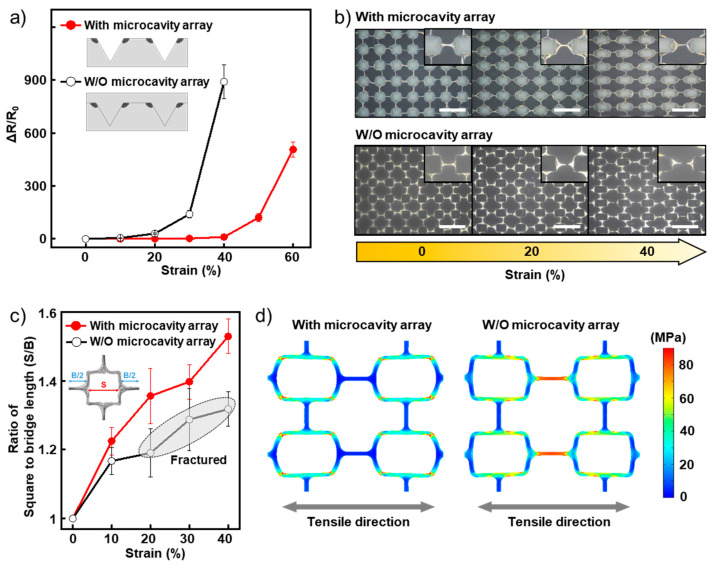
(**a**) Relative resistance change (ΔR/R_0_) in the stretchable electrodes with or without microcavity array under stretching. (**b**) Optical microscopic images of stretchable electrodes during stretching. Scale bar: 30 μm. Insets are magnified images of bridge regions at each strain value. (**c**) Changes in the ratio of the square to bridge length according to the strain. (**d**) Simulation data of the stretchable electrodes with and without microcavity array under 30% strain.

We employed a BSS bundle mesh as the conducting component in a stretchable electrode. As shown in Figure S4, the BSS bundle mesh is more efficient for deformation than a conventional mesh-shaped structure because the structure delocalizes the concentrated stress by the deformation of the square region; consequently, the structure allows the stress to be distributed more evenly. To determine the stretchability of the electrode, the relative resistance change (ΔR/R_0_) with the tensile strain was measured. Notably, an inverted pyramidal microcavity array exists on the surface of the stretchable electrode, which is naturally created by demolding during the fabrication process. As shown in Figure 3a (solid circle), the value of ΔR/R_0_ was only 10.3 with a strain of 40%. This high stretchability was also confirmed by the optical microscopic images of the stretchable electrodes taken under stretching (Figure 3b). A noticeable fracture of the BSS bundle could not be found up to 40% strain, and only a few were observed above a strain of 50% (Figure S5).

To study the causes of the high stretchability in depth, we prepared a stretchable electrode without a microcavity array on its surface. In order to fill up the microcavity array, we conducted over-coating of TPU solution on the stretchable electrode with a microcavity array. It exhibited lower stretchability and an even larger ΔR/R_0_ increment than with the microcavity array, as shown in Figure 3a (hollow circle). Accordingly, many fractures of AgNW bundles were observed even at strains as low as 20% (Figure 3b). This difference in stretchability between the cases with and without the microcavity array is explained as follows. In addition to the contribution of the BSS mesh structure, the microcavity located inside the square region of AgNW bundle mesh serves to concentrate the tensile strain applied to the stretchable electrode to the square region which is easily deformed against tensile strain, thereby preventing and mitigating the concentration of stress at bridge region. This is because the thickness of the TPU substrate under the microcavity is thinner than the region without the microcavity. (Figure 3c,d). From these results, we conclude that the high stretchability of the stretchable electrode is due to the synergistic effect of the BSS bundle mesh structure and the microcavity array located in the square region of the BSS mesh.

**Figure 4:**
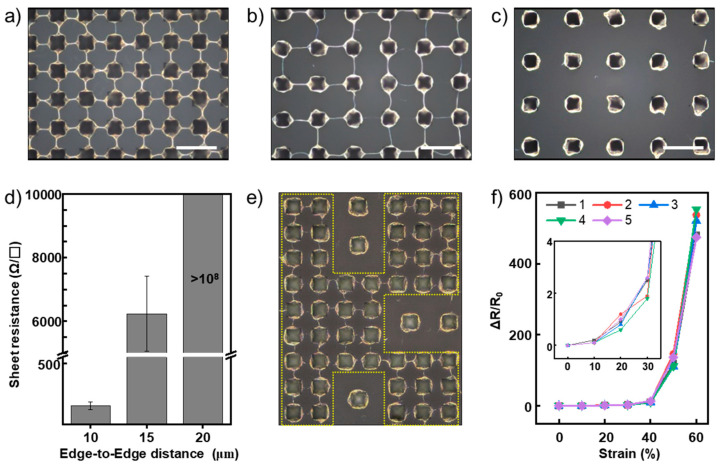
(**a**–**c**) Optical microscope images of AgNW deposited on microstructured substrates with various edge-to-edge distances between microstructures. Scale bar: 30 μm; (**a**) 10 μm; (**b**) 15 μm; (**c**) 20 μm. (**d**) Sheet resistances of the AgNW-deposited substrates shown in Figure 4a–c. In the case of the sample with an edge-to-edge distance of 20 μm, the sheet resistance value exceeded 10^8^ Ω/□. The sheet resistance is the mean value of 5 samples, and the error bar indicates standard deviation. (**e**) Image of the letter “K” composed of the BSS bundle mesh developed by self-patterning characteristics. (**f**) Relative resistance change (ΔR/R_0_) of 5 stretchable electrodes fabricated by repeatedly using the same microstructured PDMS substrate.

To investigate the effect of the distance between microstructures on the formation of AgNW bundle mesh, we spray coated AgNW dispersions on microstructured PDMS substrates with various distances between adjacent structures of 10, 15, and 20 μm. As shown in Figure 4a–c, as the distance between adjacent microstructures increased, the width and number of bridges in the AgNW bundle mesh decreased, and no bridge connecting neighboring squares was formed even at a distance of 20 μm. It seems that the liquid bridges with a distance of 20 μm were not sustained because of the balance between the structural cohesive force and the capillary force [35,39]. Consistent with this observation, the sheet resistance sharply increased as the distance between the microstructures increased, reaching above 10^8^ Ω/□ at a distance of 20 μm (Figure 4d). These results indicate that if only the microstructured substrate—the target substrate of the spray coating—is deliberately designed by considering the self-patterning ability (i.e., deliberate electrical isolation by adjusting the distance between microstructures), it is possible to easily and repeatedly produce intricately patterned stretchable electrodes without additional patterning processes, such as photolithography and laser ablation. To demonstrate the self-patterning ability, we prepared the microstructured substrates that formed AgNW bundle mesh according to the shape of the letter “K”. The distance between the microstructures inside the letter was 10 μm, and that outside the letter was 20 μm. After spraying the AgNWs dispersion, the deposited AgNWs were transferred to the TPU substrate. As shown in Figure 4e, the BSS bundle mesh was only formed inside the character and not outside the character.

After peeling off the stretchable electrode, the microstructured substrate returned to its initial state without the AgNW or TPU residue as before the AgNW was spray coated (Figure S6a,b). From these observations, we fabricated five stretchable electrodes by repeatedly using the same microstructured substrate. As a result, the fabricated stretchable electrodes exhibited high reproducibility (Figure 4f), which means that the fabrication strategy presented in this work is applicable to continuous low-cost production when applied to roll-to-roll processes.

## 4. Conclusions

In this paper, we present a self-patterned stretchable electrode by liquid bridge evaporation. In the proposed process, the stretchable electrode is composed of a BSS AgNW bundle mesh embedded in a TPU matrix with a microcavity array. A regular and uniform bundle mesh is developed by the formation and evaporation of a liquid bridge located between microstructures, and the microcavity array on the surface of the TPU matrix is naturally created by demolding. Owing to the synergistic effect of the bridged square-shaped bundle mesh and microcavity array, the stretchable electrode in our experiments exhibited high stretchability with a low ΔR/R_0_ of 10.3 at a strain of 40%. Furthermore, by exploiting the self-patterning ability based on the difference in the ability to form liquid bridges according to the distance between microstructures, we successfully demonstrated a stretchable AgNW bundle mesh with complex patterns without using additional patterning processes. In particular, stretchable electrodes were fabricated by spray coating and bar coating, which are widely used in industry for low-cost mass production.

## Data Availability

The study did not report any data.

## Supplementary Materials

The following are available online at https://www.mdpi.com/article/10.3390/nano11112865/s1, Figure S1: FE-SEM Images of surface-treated PDMS substrate by UVO. Figure S2: XPS Data of the surface treated-PDMS substrates, Figure S3: Images and contact angles of static droplets on pristine and surface treated PDMS substrate, Figure S4: FEM Simulation results of the stress distribution of conventional mesh structure and the BSS mesh structure under 30% strain, Figure S5: Optical microscopy images of the stretchable electrode stretched up to 70% tensile strain, Figure S6: FE-SEM Images of microstructured PDMS substrates before spray coating and after peeling off the stretchable electrode.

## References

[1] Son D., Lee J., Qiao S., Ghaffari R., Kim J., Lee J.E., Song C., Kim S.J., Lee D.J., Jun S.W. (2014). Multifunctional wearable devices for diagnosis and therapy of movement disorders. Nat. Nanotechnol..

[2] Lee H., Choi T.K., Lee Y.B., Cho H.R., Ghaffari R., Wang L., Choi H.J., Chung T.D., Lu N., Hyeon T. (2016). A graphene-based electrochemical device with thermoresponsive microneedles for diabetes monitoring and therapy. Nat. Nanotechnol..

[3] Yokota T., Zalar P., Kaltenbrunner M., Jinno H., Matsuhisa N., Kitanosako H., Tachibana Y., Yukita W., Koizumi M., Someya T. (2016). Ultraflexible organic photonic skin. Sci. Adv..

[4] Kim J., Shim H.J., Yang J., Choi M.K., Kim D.C., Kim J., Hyeon T., Kim D.-H. (2017). Ultrathin quantum dot display integrated with wearable electronics. Adv. Mater..

[5] Choi M.K., Yang J., Kim D.C., Dai Z., Kim J., Seung H., Kale V.S., Sung S.J., Park C.R., Lu N. (2018). Extremely vivid, highly transparent, and ultrathin quantum dot light-emitting diodes. Adv. Mater..

[6] Xu L., Gutbrod S.R., Bonifas A.P., Su Y., Sulkin M.S., Lu N., Chung H.-J., Jang K.-I., Liu Z., Ying M. (2014). 3D multifunctional integumentary membranes for spatiotemporal cardiac measurements and stimulation across the entire epicardium. Nat. Commun..

[7] Park S., Heo S.W., Lee W., Inoue D., Jiang Z., Yu K., Jinno H., Hashizume D., Sekino M., Yokota T. (2018). Self-powered ultra-flexible electronics via nano-grating-patterned organic photovoltaics. Nature.

[8] Wonryung L., Shingo K., Masase N., Yasutoshi J., Itsuro S., Yusuke I., Tomoyuki Y., Masaki S., Malliaras G.G., Tomoyuki Y. (2018). Nonthrombogenic, stretchable, active multielectrode array for electroanatomical mapping. Sci. Adv..

[9] Kim Y., Kweon O.Y., Won Y., Oh J.H. (2019). Deformable and Stretchable Electrodes for Soft Electronic Devices. Macromol. Res..

[10] Matsuhisa N., Chen X., Bao Z., Someya T. (2019). Materials and structural designs of stretchable conductors. Chem. Soc. Rev..

[11] Hu M., Cai X., Guo Q., Bian B., Zhang T., Yang J. (2016). Direct pen writing of adhesive particle-free ultrahigh silver salt-loaded composite ink for stretchable circuits. ACS Nano.

[12] Yue W., Chenxin Z., Raphael P., Hongping Y., Lihua J., Shucheng C., Francisco M.-L., Franziska L., Jia L., Rabiah L.N. (2017). A highly stretchable, transparent, and conductive polymer. Sci. Adv..

[13] Wang J., Cai G., Li S., Gao D., Xiong J., Lee P.S. (2018). Printable superelastic conductors with extreme stretchability and robust cycling endurance enabled by liquid-metal particles. Adv. Mater..

[14] Zhao Y., Huang X. (2017). Mechanisms and materials of flexible and stretchable skin sensors. Micromachines.

[15] Zhang C., Khan A., Cai J., Liang C., Liu Y., Deng J., Huang S., Li G., Li W.-D. (2018). Stretchable transparent electrodes with solution-processed regular metal mesh for an electroluminescent light-emitting film. ACS Appl. Mater. Interfaces.

[16] Guo C.F., Liu Q., Wang G., Wang Y., Shi Z., Suo Z., Chu C.-W., Ren Z. (2015). Fatigue-free, superstretchable, transparent, and biocompatible metal electrodes. Proc. Natl. Acad. Sci. USA.

[17] Ko E.-H., Kim H.-J., Lee S.-M., Kim T.-W., Kim H.-K. (2017). Stretchable ag electrodes with mechanically tunable optical transmittance on wavy-patterned PDMS substrates. Sci. Rep..

[18] Tang J., Guo H., Zhao M., Yang J., Tsoukalas D., Zhang B., Liu J., Xue C., Zhang W. (2015). Highly stretchable electrodes on wrinkled polydimethylsiloxane substrates. Sci. Rep..

[19] Xu R., Zverev A., Hung A., Shen C., Irie L., Ding G., Whitmeyer M., Ren L., Griffin B., Melcher J. (2018). Kirigami-inspired, highly stretchable micro-supercapacitor patches fabricated by laser conversion and cutting. Microsyst. Nanoeng..

[20] Bao Y., Hong G., Chen Y., Chen J., Chen H., Song W.-L., Fang D. (2020). Customized kirigami electrodes for flexible and deformable lithium-ion batteries. ACS Appl. Mater. Interfaces.

[21] Hong J.-Y., Kim W., Choi D., Kong J., Park H.S. (2016). Omnidirectionally stretchable and transparent graphene electrodes. ACS Nano.

[22] Hong S., Lee J., Do K., Lee M., Kim J.H., Lee S., Kim D.-H. (2017). Stretchable electrode based on laterally combed carbon nanotubes for wearable energy harvesting and storage devices. Adv. Funct. Mater..

[23] Cho S., Kang D., Lee H., Kim M.P., Kang S., Shanker R., Ko H. (2021). Highly stretchable sound-in-display electronics based on strain-insensitive metallic nanonetworks. Adv. Sci..

[24] Liu H.-S., Pan B.-C., Liou G.-S. (2017). Highly transparent AgNW/PDMS stretchable electrodes for elastomeric electrochromic devices. Nanoscale.

[25] Kumar A., Shaikh M.O., Chuang C.-H. (2021). Silver nanowire synthesis and strategies for fabricating transparent conducting electrodes. Nanomaterials.

[26] Liu G.-S., Liu C., Chen H.-J., Cao W., Qiu J.-S., Shieh H.-P.D., Yang B.-R. (2016). Electrically robust silver nanowire patterns transferrable onto various substrates. Nanoscale.

[27] Liang J., Li L., Chen D., Hajagos T., Ren Z., Chou S.-Y., Hu W., Pei Q. (2015). Intrinsically stretchable and transparent thin-film transistors based on printable silver nanowires, carbon nanotubes and an elastomeric dielectric. Nat. Commun..

[28] Xiong J., Li S., Ye Y., Wang J., Qian K., Cui P., Gao D., Lin M.-F., Chen T., Lee P.S. (2018). A deformable and highly robust ethyl cellulose transparent conductor with a scalable silver nanowires bundle micromesh. Adv. Mater..

[29] Xiong J., Li S., Ciou J.-H., Chen J., Gao D., Wang J., Lee P.S. (2021). A tailorable spray-assembly strategy of silver nanowires-bundle mesh for transferable high-performance transparent conductor. Adv. Funct. Mater..

[30] Kim C.H., Lee D.H., Youn J., Lee H., Jeong J. (2021). Simple and cost-effective microfabrication of flexible and stretchable electronics for wearable multi-functional electrophysio-logical monitoring. Sci. Rep..

[31] Niu S., Matsuhisa N., Beker L., Li J., Wang S., Wang J., Jiang Y., Yan X., Yun Y., Burnett W. (2019). A wireless body area sensor network based on stretchable passive tags. Nat. Electron..

[32] Zhu J., Hu Z., Song C., Yi N., Yu Z., Liu Z., Liu S., Wang M., Dexheimer M.G., Yang J. (2021). Stretchable wideband dipole antennas and rectennas for RF energy harvesting. Mater. Today Phys..

[33] Bae G.Y., Han J.T., Lee G., Lee S., Kim S.W., Park S., Kwon J., Jung S., Cho K. (2018). Pressure/temperature sensing bimodal electronic skin with stimulus discriminability and linear sensitivity. Adv. Mater..

[34] Lee G., Lee S.G., Chung Y., Bae G.Y., Lee S., Ryu S., Cho K. (2016). Omnidirectionally and highly stretchable conductive electrodes based on noncoplanar zigzag mesh silver nanowire arrays. Adv. Electron. Mater..

[35] Wu Y., Su B., Jiang L. (2012). Smartly aligning nanowires by a stretching strategy and their application as encoded sensors. ACS Nano.

[36] Vakarelski I.U., Chan D.Y.C., Nonoguchi T., Shinto H., Higashitani K. (2009). Assembly of gold nanoparticles into microwire networks induced by drying liquid bridges. Phys. Rev. Lett..

[37] Vakarelski I.U., Kwek J.W., Tang X., O’Shea S.J., Chan D.Y.C. (2009). Particulate templates and ordered liquid bridge networks in evaporative lithography. Langmuir.

[38] Lee G., Bae G.Y., Son J.H., Lee S., Kim S.W., Kim D., Lee S.G., Cho K. (2020). User-interactive thermotherapeutic electronic skin based on stretchable thermochromic strain sensor. Adv. Sci..

[39] Yan C., Su B., Shi Y., Jiang L. (2019). Liquid bridge induced assembly (LBIA) strategy: Controllable one-dimensional patterning from small molecules to macro-molecules and nanomaterials. Nano Today.

