# Supplementary material for: Self-Patterned Stretchable Electrode Based on Silver Nanowire Bundle Mesh Developed by Liquid Bridge Evaporation
Source: Nanomaterials (Basel). 2021 Oct 27;11(11):2865. doi: 10.3390/nano11112865 (PMC8621255; doi:10.3390/nano11112865)
Supplement: Supplementary file 1 [file nanomaterials-11-02865-s001.zip › nanomaterials-1426104-supplementary.pdf]

nanomaterials

Supplementary Materials

Self-Patterned Stretchable Electrode Based on Silver Nanowire Bundle Mesh developed by Liquid Bridge Evaporation

Eun Young An [1,2,†], Siyoung Lee [3,†], Seung Goo Lee [4], Eunho Lee [5], Jeong Ju Baek [1], Gyojic Shin [1], Kyung Ho Choi [1], Jeong Ho Cho [2,*] and Geun Yeol Bae [1,*]

[1] Green and Sustainable Materials R&D Department, Korea Institute of Industrial Technology, Cheonan 31056, Korea; dksdmsdud97@kitech.re.kr (E.Y.A.); baekjj@kitech.re.kr (J.J.B.); gyshin@kitech.re.kr (G.S.); khchoi@kitech.re.kr (K.H.C.)
[2] Department of Chemical and Biomolecular Engineering, Yonsei University, Seoul 03722, Korea
[3] Department of Chemical Engineering, Pohang University of Science and Technology, Pohang 37673, Korea; challenge@postech.ac.kr
[4] Department of Chemistry, University of Ulsan, Ulsan 44610, Korea; lees9@ulsan.ac.kr
[5] Department of Chemical Engineering, Kumoh National Institute of Technology, Gumi 39177, Korea; leeeh@kumoh.ac.kr
* Correspondence: jhcho94@yonsei.ac.kr (J.H.C.); gybae@kitech.re.kr (G.Y.B.)
† These authors contributed equally to this work.

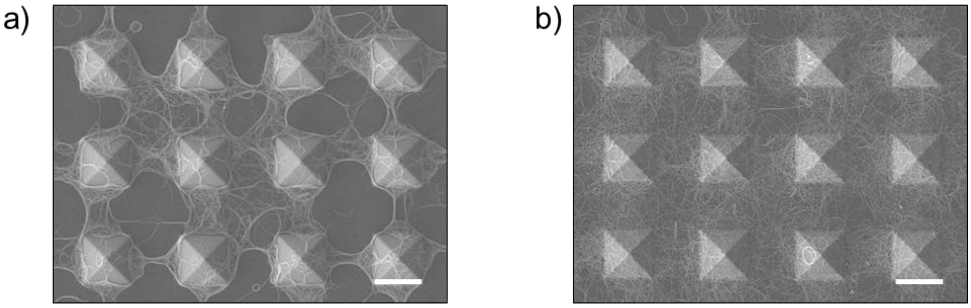

Figure S1. FE-SEM Images of surface-treated PDMS substrate by UVO. Scale bar: 10 μm; (**a**) for 10 min; (**b**) for 60 min.

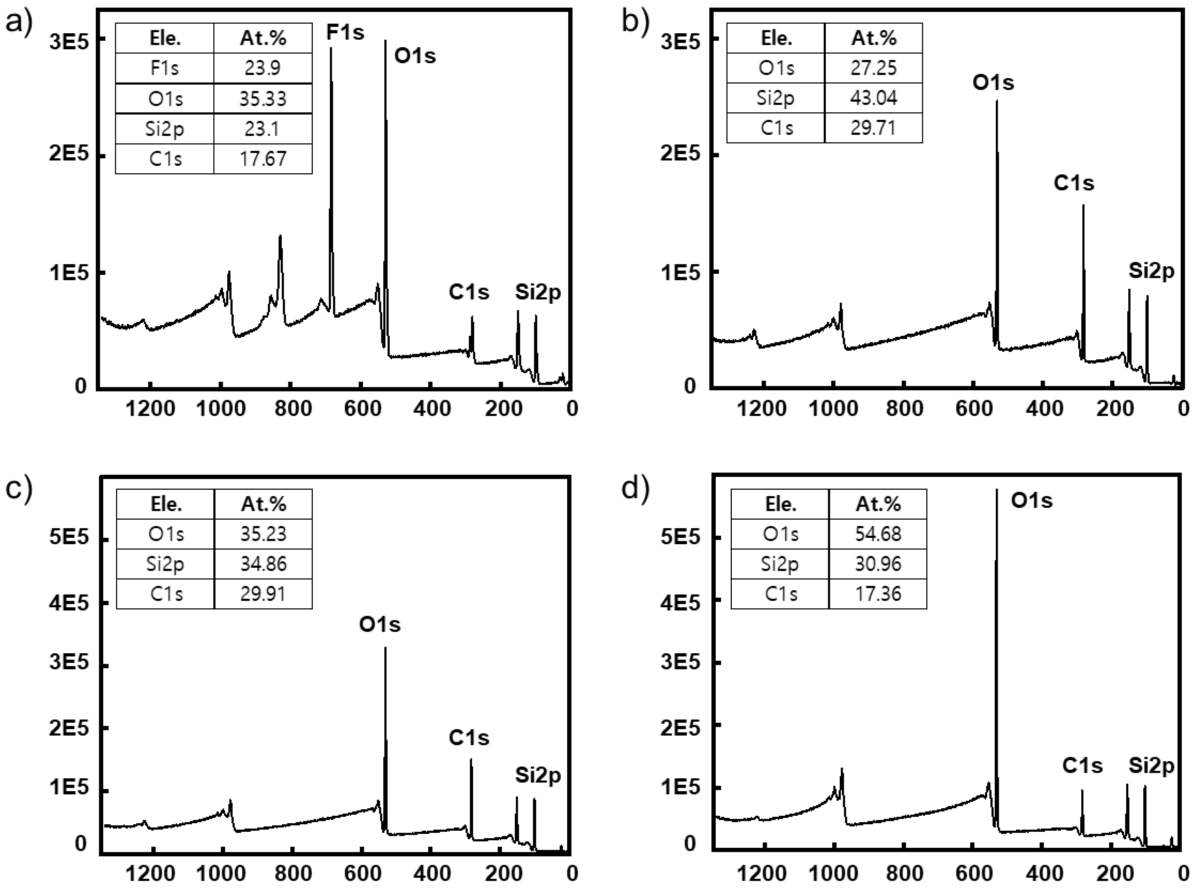

Figure S2. XPS Data of the surface treated-PDMS substrates; (**a**) FOTS-treated; (**b**) Pristine; (**c**) UVO treated for 10 min; (**d**) UVO treated for 60 min.

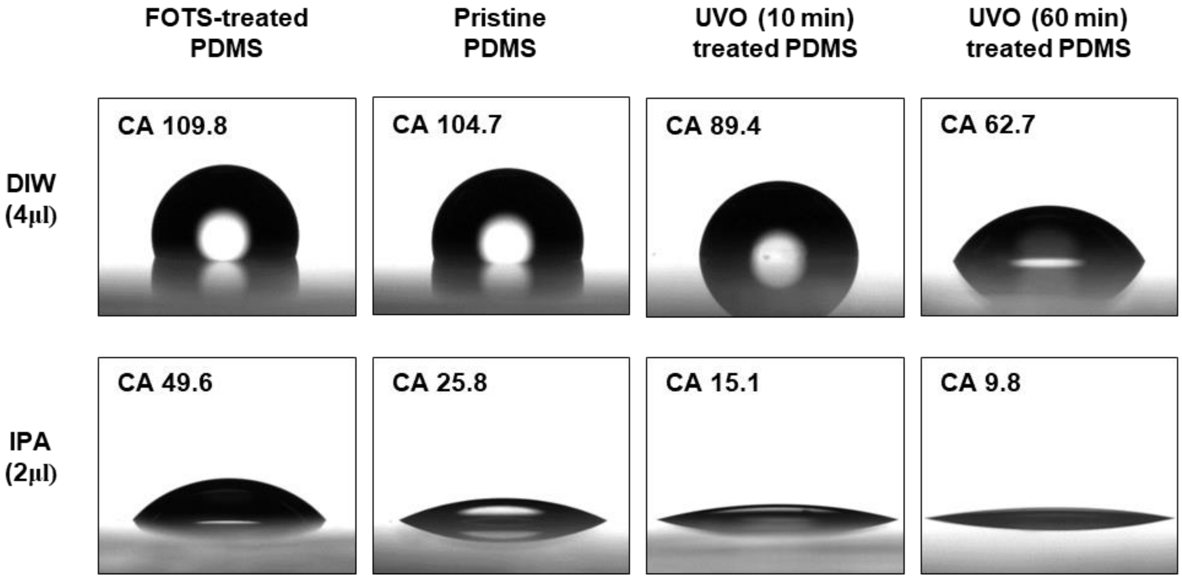

Figure S3. Images and contact angles of static droplets on pristine and surface treated PDMS substrate. The substrates are flat, not microstructured.

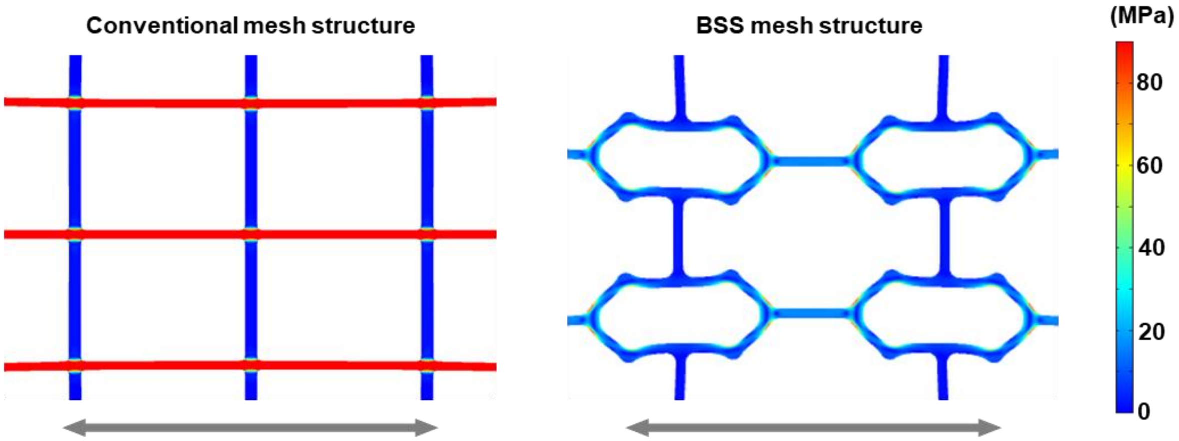

Figure S4. FEM Simulation results of the stress distribution of conventional mesh structure and the BSS mesh structure under 30% strain. The simulation was carried out under the assumption that there is only a mesh structure without a substrate.

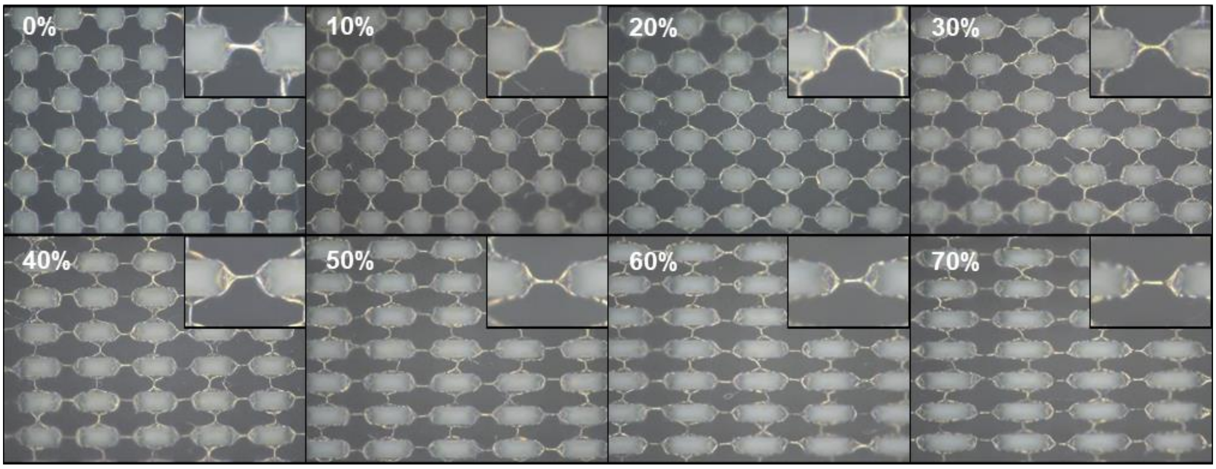

Figure S5. Optical microscopy images of the stretchable electrode stretched up to 70% tensile strain.

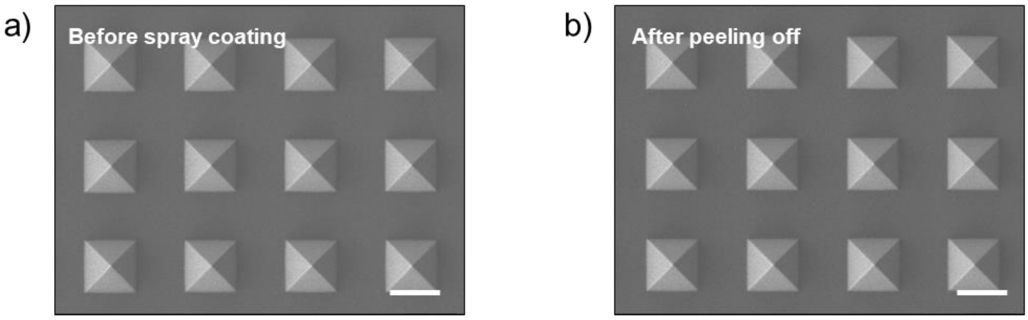

Figure S6. (**a**, **b**) FE-SEM Images of microstructured PDMS substrates before spray coating and after peeling off the stretchable electrode, respectively.
